# Robust Laminated Anode with an Ultrathin Titanium Nitride Layer for High-Efficiency Top-Emitting Organic Light-Emitting Diodes

**DOI:** 10.3390/molecules27175723

**Published:** 2022-09-05

**Authors:** Jia-Heng Cai, Qi-Sheng Tian, Xiao-Zhao Zhu, Zhi-Hao Qu, Wei He, Dong-Ying Zhou, Liang-Sheng Liao

**Affiliations:** 1 Institute of Functional Nano & Soft Materials, Jiangsu Key Laboratory for Carbon-Based Functional Materials & Devices, Soochow University, Suzhou 215123, China; 2 Macao Institute of Materials Science and Engineering, Macau University of Science and Technology, Taipa 999078, Macau, China

**Keywords:** organic light-emitting diodes, bottom reflective anode, titanium nitride, complementary metal-oxide-semiconductor, microdisplays

## Abstract

The effective reflective anode remains a highly desirable component for the fabrication of reliable top-emitting organic light-emitting diodes (TE-OLEDs) which have the potential to be integrated with complementary metal-oxide-semiconductor (CMOS) circuits for microdisplays. This work demonstrates a novel laminated anode consisting of a Cr/Al/Cr multilayer stack. Furthermore, we implement an ultra-thin titanium nitride (TiN) layer as a protective layer on the top of the Cr/Al/Cr composite anode, which creates a considerably reflective surface in the visible range, and meanwhile improves the chemical stability of the electrode against the atmosphere or alkali environment. Based on [2-(2-pyridinyl-N)phenyl-C](acetylacetonate)iridium(III) as green emitter and Mg/Ag as transparent cathode, our TE-OLED using the TiN-coated anode achieves the maximum current efficiency of 71.2 cd/A and the maximum power efficiency of 66.7 lm/W, which are 81% and 90% higher than those of the reference device without TiN, respectively. The good device performance shows that the Cr/Al/Cr/TiN could function as a promising reflective anode for the high-resolution microdisplays on CMOS circuits.

## 1. Introduction

Microdisplays featuring high resolution and aperture ratios of emitting pixels have the potential to revolutionize the near-to-eye applications ranging from healthcare and military to personal entertainment [1,2,3,4,5,6]. The first commercial microdisplay, which combined inorganic light-emitting diode backlights and liquid crystal displays [7,8,9], had a relatively low field angle and a relatively small field of view, making them unsuitable for various applications. Fortunately, organic light-emitting diodes (OLEDs) have been emerging as the emitting pixel of microdisplays due to their advantages in high contrast, fast response, and low-temperature effect [10,11,12,13]. A feasible way to maximize the display resolution comes from integrating OLEDs with the circuits utilizing complementary metal-oxide-semiconductor (CMOS) transistors on Si [14,15]. Since the opaque Si-based circuits generally require top-emitting OLEDs (TE-OLEDs), the bottom anode is therefore one of the most critical components in the TE-OLEDs. The anode should meet with high optical reflectivity, low electrical resistivity, and suitable work function for efficient injection of holes [16,17,18]. In addition, the anodes may be exposed to the ambient before the fabrication of TE-OLEDs on top since they are commonly provided from a separated pre-process in the CMOS foundry [19,20]. Therefore, the chemical stability and CMOS-process compatibility of the anodes should also be carefully considered for the preparation of efficient TE-OLEDs.

Over the years, researchers have explored various materials for the anode of TE-OLEDs, including Al, Ag, Ni, Au, etc. [16,17,18,21,22]. Among them, Al and Ag have considerably high reflectivity (typically above 90%), which makes them suitable for the electrode. However, the work function of Al and Ag are generally around 4.3 eV, which is not high enough to inject holes to the highest occupied molecular orbitals (HOMOs) of the adjacent hole-transporting layers (HTLs). This could be addressed by using post-treatments further to improve the work function of Al and Ag [16,21,23,24,25]. For instance, Wang et al. employed Al/Ni to replace Al and obtained a work function approaching 5.2 eV [21]. Lee et al. reported using O_2_ plasma treatment to increase the work function of Ag anode by 0.4 eV and successfully decreased the turn-on voltage of TE-OLEDs from 17 to 7 V [23]. On the other hand, since Ag has less process compatibility with the CMOS foundry, Al seems to be one auspicious material as the anode of TE-OLEDs. However, Al is very sensitive to oxygen and easy to be oxidized in the ambient. Therefore, it is still urgent to develop high-work function and chemically stable anodes based on the Al metal. 

Owing to its unique properties, such as high hardness and melting temperature (2900 °C), titanium nitride (TiN) is promising in many applications ranging from the coating of cutting tools to diffusion barriers of microelectronic devices [26,27,28]. In this work, we designed a new silicon-based anode structure for green TE-OLEDs. The anode is composed of Cr/Al/Cr/TiN, which has good reflectivity, high work function, and excellent chemical stability. Using Cr/Al/Cr/TiN as the bottom anode enables the accomplishment of highly efficient TE-OLEDs, showing its great potential for industrial applications.

## 2. Materials and Methods

For the fabrication of TE-OLEDs, the SiO_2_ substrates used were cleaned with deionized water and ethanol for 10 min each time. Followed by drying in an oven, the substrates were treated in an ultraviolet ozone ambient for 15 min for further removing the contaminations on the surface. Then, the as-designed laminated anodes were deposited by magnetron sputtering under a vacuum of 10^−3^ Pa. A 5-nm-thick Cr layer was firstly prepared onto the substrate under the Ar atmosphere with a pressure of 2 Pa, using a DC power of 200 W. A 35-nm-thick Al layer was then prepared on the surface of the Cr layer under the same Ar atmosphere with an RF power of 200 W. A second Cr layer was deposited as its first layer. TiN was formed by adjusting the pressure to 2 Pa under the Ar and N_2_ atmosphere with a DC power supply of 200 W. For comparison, a Mo-coated Cr/Al/Cr/Mo electrode was prepared by depositing a 5-nm-thick Mo layer onto the Cr/Al/Cr under the Ar atmosphere with a pressure of 2 Pa, using a DC power of 200 W. Note that these layers were deposited through a shadow mask, the patterned Cr/Al/Cr, Cr/Al/Cr/TiN, and Cr/Al/Cr/Mo were therefore formed on the quartz substrate.

Then, the top-emitting OLED devices were prepared in a FS-450 thermal evaporator (Suzhou Fangsheng, Suzhou, China) under the vacuum condition with the basic pressure below 4.0 × 10^−4^ Pa. After the patterned substrate was transferred to the vacuum chamber, organic, and metallic layers were sequentially deposited with the evaporation rates of 0.5~5.0 Å/s. The emitting area of TE-OLED devices was defined as 9 mm^2^ by the cross section of the electrodes. 

To conducting the alkali-resistant test, 0.5 g of NaCO_3_ powder was added to 80 mL of deionized water, and the solution was stirred with a glass rod to dissolve the powder. The pH test paper was used to test the alkalinity of the solution, which was controlled at about 10. The Cr/Al/Cr/TiN and Cr/Al/Cr/Mo coated substrates with different variables were soaked in an alkaline solution. Then they were put into an ultrasonic cleaner for ultrasonic treatment, with the temperature maintained at 50 degrees and the frequency at 100 Hz.

Film morphologies were examined by G500 field emission scanning electron microscope (Carl Zeiss, Oberkochen, Germany) and atomic force microscope (Asylum Research, Oxford Instruments, Abingdon, UK). The reflectivity was determined used a Lambda 950 UV-vis-NIR spectrophotometer (Perkin Elmer Inc., Waltham, MA, USA). The sheet resistance of the electrodes was measured by a ST2258A four-point probe method (Jingge, Suzhou, China). The ultraviolet photoelectron spectroscopy (UPS) spectra were obtained on a ESCALAB 250Xi photoelectron spectrometer (ThermoFisher Scientific, Waltham, MA, USA) using HeI irradiation with hν = 21.22 eV. The value of WF is determined from the secondary electron cutoff using the relation $WF=h\nu -\left(E_{F}-E_{cutoff}\right)$, where hν, $E_{F}$, and $E_{cutoff}$ are the photon energy of the excitation light (21.22 eV), the Fermi level edge, and the measured secondary electron cutoff, respectively. The current density–voltage–luminance characteristics and EL spectra of unpackaged OLED were measured simultaneously by a computer-controlled programmable Model 2400 power source (Keithley, Beaverton, OR, USA) and a PR 655 Spectra Scan (Photo Research Inc., Chatsworth, GA, USA).

## 3. Results and Discussion

**Figure 1:**
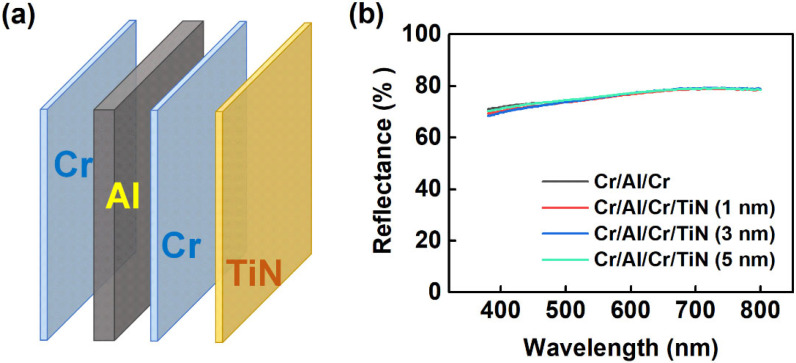
(**a**) Schematic structure of the composite anode; (**b**) Reflectance spectra of the Cr/Al/Cr/TiN anodes with different thicknesses of TiN.

In this work, we design and fabricate a considerably reflective anode for the TE-OLEDs, which has the potential to be integrated with the CMOS circuits for microdisplays. The anode consists of a laminated structure including Cr (5 nm)/Al (35 nm)/Cr (5 nm), as schemed in Figure 1a. To improve the chemical stability of the composite anode, an ultrathin TiN layer with different thicknesses (i.e., 1, 3, and 5 nm) is coated on the surface of top Cr layer as a protective layer. The previous work has reported the Cr/Al/Cr as a promising anode for TE-OLEDs [29,30]. However, the chemical stability and hole injection effectiveness of the Cr/Al/Cr were yet addressed. Atomic force microscope (AFM) characterization reveals the TiN forms a smooth and pinhole-free film on the surface of Cr/Al/Cr due to the addition of the Cr adhesive layer (Supplementary Figure S1 in the Supporting Information). The root-mean-square roughness (R_q_) of composite anode reduces with the increase of TiN thickness and is determined to be 1.63 nm as 5 nm TiN is used. The smoothened surface due to the addition of TiN may alleviate the electric field of point discharge and significantly avoid the short circuit issue in OLEDs [31]. 

Effects of the TiN coating on the optical and electric properties of composite anode were also studied. As shown in Figure 1b, the multilayer stacks without and with TiN show almost identical reflectance spectra, with the relatively high reflectance of 70~80% over the visible light region. In terms of electric property (seen in Supplementary Figure S2), the sheet resistances of the composite cathodes with 1, 3, and 5 nm TiN are determined to be 5.1, 6.4, and 8.8 Ω/☐, respectively. The results imply that the addition of TiN can retain a high reflectance and a low sheet resistance for the metallic multilayers, which indicates a minimal photonic or electric loss when incident light or charge current goes through the Cr/Al/Cr/TiN layer, thus ensuring its applicability as an anode material.

**Figure 2:**
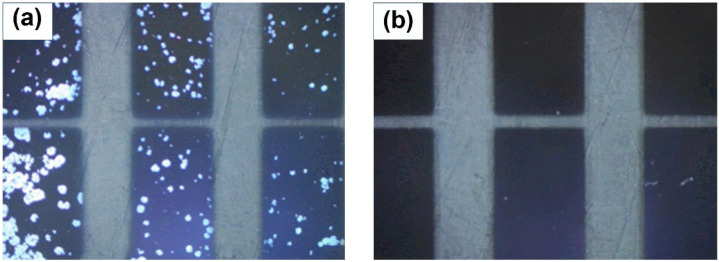
Optical images of the Cr/Al/Cr/TiN anodes (**a**) without and (**b**) with TiN after being stored in the ambient for 100 h.

To assess the effectiveness of TiN in protecting the active metallic electrodes, an aging test was conducted by storing the as-prepared Cr/Al/Cr and Cr/Al/Cr/TiN (5 nm) films in an ambient with a relative humidity of 85% and a temperature of 85 °C. Figure 2 shows the optical microscope images of the samples after 100 h of storage. As anticipated, the Cr/Al/Cr seems to be corroded in the ambient, which is evidenced by the bright spots in the optical microscope image. In contrast, the Cr/Al/Cr/TiN film after the same aging test remains a clear and uniform pattern, suggesting the coating of TiN could form a barrier to protect the Cr and Al from corrosion by oxygen or moisture.

**Figure 3:**
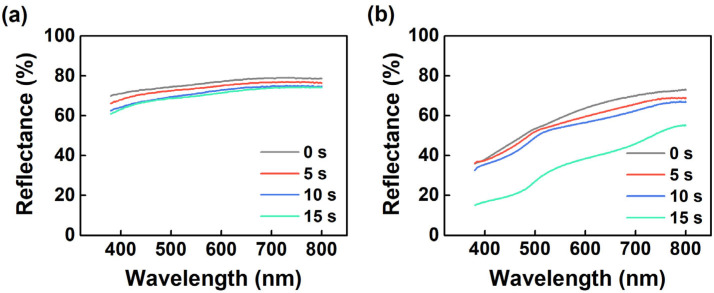
Reflectance spectra of the (**a**) Cr/Al/Cr/TiN and (**b**) Cr/Al/Cr/Mo anodes under the ultrasonic treatment for a different time.

In most manufacturing processes the substrate cleaning generally proceeds in an alkaline environment. Therefore, we performed the alkali-resistant test of the Cr/Al/Cr/TiN anode by subjecting the sample to ultrasonic treatment in a sodium bicarbonate solution (pH = 10). To make a comparison, another electrode composited of Cr/Al/Cr/Mo with 5 nm Mo was fabricated and tested in the same manner as Cr/Al/Cr/TiN. Figure 3 shows the reflectance spectra of these two films before and after the different duration of ultrasonic treatment. After being treated for 15 s, the reflectance of the Cr/Al/Cr/TiN drops from 75.2% to 69.3% at a wavelength of 530 nm, whereas for the Cr/Al/Cr/Mo the reflectance drops from 56.6% to 32.2%. We also measured the sheet resistance of the composite electrodes before and after the ultrasonic treatment, as shown in Table 1. Noticed that the sheet resistances of Cr/Al/Cr/TiN are much lower than those of Cr/Al/Cr/Mo, since Mo has quite a low electrical resistivity of ca. 5 μΩ·cm due to its metallic nature, while TiN is generally considered as wide energy gap material with a relative high resistivity of 21.7 μΩ·cm [26]. However, both absolute values of sheet resistance for Cr/Al/Cr/Mo and Cr/Al/Cr/TiN are far below the general requirement of electrodes used for OLEDs (~30 Ω/□). After the treatment for 15 s, the sheet resistance of the Cr/Al/Cr/TiN electrode only increases by 27%, while that of the Cr/Al/Cr/Mo electrode increases by 126%. Clearly, the ultrasonic treatment results in the degradation of both composite electrodes reflected by the reduction of reflectance and increase of sheet resistance. However, the changes in reflectance and sheet resistance of Cr/Al/Cr/TiN are much smaller than those of Cr/Al/Cr/Mo, indicating the alkali-resistant property of the composite electrodes is successfully improved by the TiN layer.

**Figure 4:**
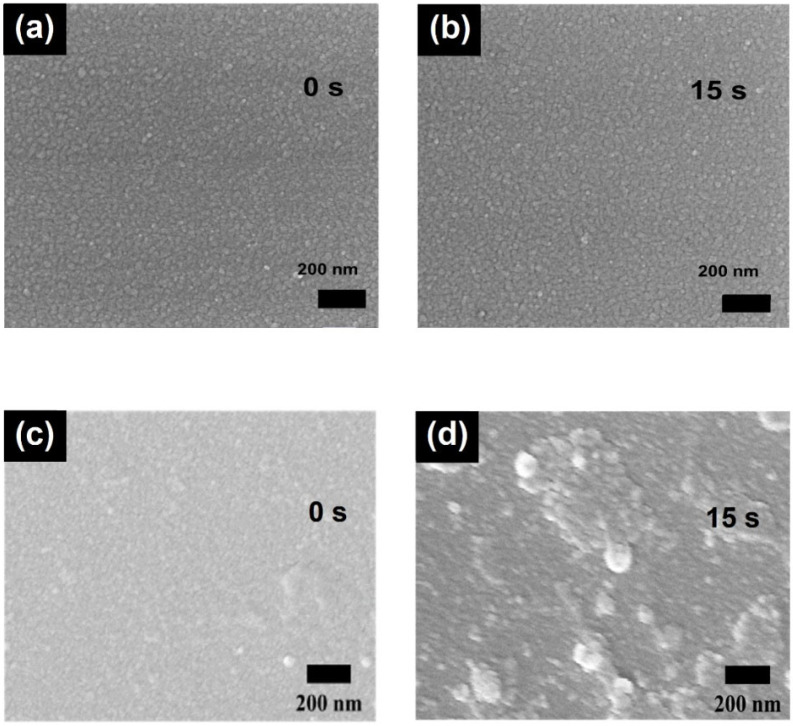
SEM images of the Cr/Al/Cr/TiN anode under the ultrasonic treatment for (**a**) 0 s and (**b**) 15 s; SEM images of the Cr/Al/Cr/Mo anode under the ultrasonic treatment for (**c**) 0 s and (**d**) 15 s.

To reveal the different roles of TiN and Mo in protecting the metallic electrodes, scanning electron microscopy (SEM) was utilized to follow the changes in morphology of Cr/Al/Cr/TiN and Cr/Al/Cr/Mo during the ultrasonic treatment. As seen in Figure 4 (as well as in Supplementary Figures S3 and S4), the Cr/Al/Cr/TiN shows a similar surface profile as a function of treatment time with uniformly distributed nanometer-scale domains, whereas the Cr/Al/Cr/Mo is gradually damaged. The Cr/Al/Cr/TiN exhibits much stronger alkali-resistant than the Cr/Al/Cr/Mo. This observation could well explain the different changes in reflectance and sheet resistance of Cr/Al/Cr/TiN and Cr/Al/Cr/Mo during the alkali-resistant test. It also indicates that the TiN coating has very high corrosion resistance and is capable to protect the metallic electrode from being destructed under a harsher environment, such as the ultrasonic treatment.

**Figure 5:**
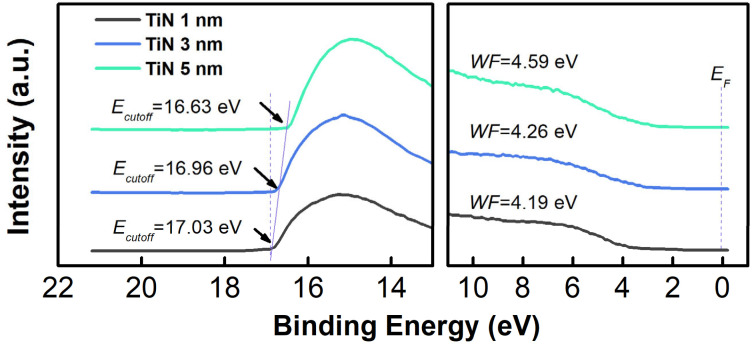
UPS spectra of the Cr/Al/Cr/TiN anodes with different thicknesses of TiN.

The work function (WF) of Cr/Al/Cr/TiN with different thicknesses of TiN was estimated by means of ultraviolet photoelectron spectroscopy (UPS) [32,33]. Figure 5 shows their photoemission spectra in the secondary electron cutoff and valence band regions. The value of WF for the Cr/Al/Cr/TiN with 1, 3, and 5 nm TiN are determined to be 4.19, 4.26, and 4.59 eV, respectively. The WF of composite anode with 5 nm TiN is comparable to those of the most anode materials (e.g., ITO~4.8 eV) [34], displaying favorable match with the HOMO level of common HTLs (5.0–5.4 eV) [35,36]. The WF of Cr and Al used in the anode are 4.19 eV and 4.27 eV respectively (Supplementary Figure S5). Since an ideal hole injection requires the HOMO level of HTL being close to the WF of the anode, in the present work the energy level alignment at the Cr/Al/Cr/TiN/HTL interfaces may facilitate the hole injection from the modified anode to the HTL. Note that the addition of HAT-CN as hole injection layer between anode and HTL layer can further improve the hole injection ability and overcome the energy barrier. In addition, considering the production cost, the designed anode structure is better than the noble metal with higher WF such as Au [37]. Thus, these results trigger the use of TiN as an interlayer between the Cr/Al/Cr anode and the HTL for efficient hole injection in OLED devices.

**Figure 6:**
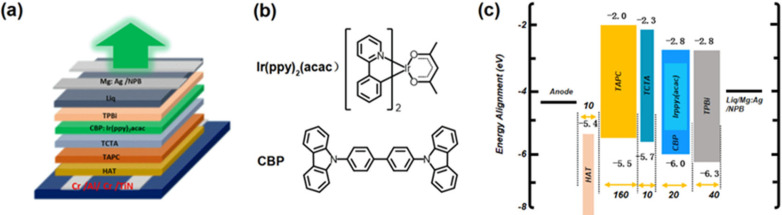
(**a**) Schematic structure of TE-OLED using the Cr/Al/Cr/TiN anode; (**b**) molecular structures of Ir(ppy)_2_(acac) and CBP; (**c**) energy band diagram of the device.

**Figure 7:**
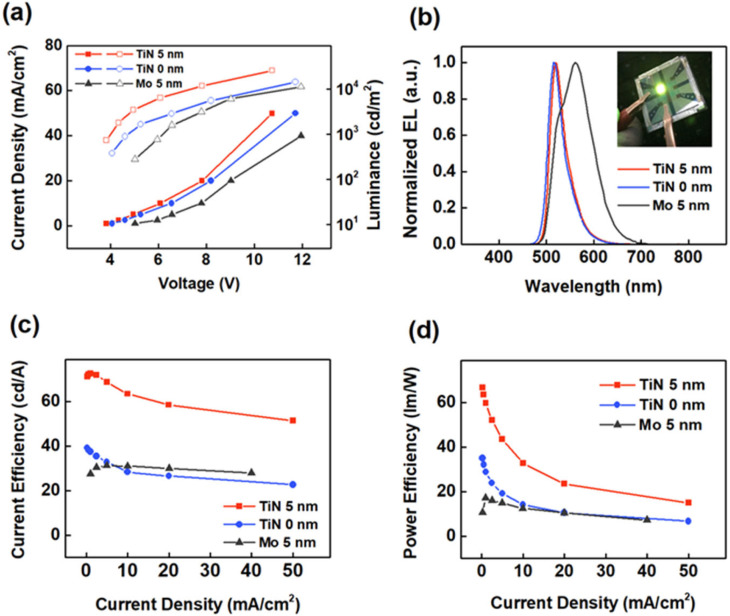
(**a**) Current density-voltage-luminance characteristics, (**b**) normalized EL spectra, (**c**) current efficiency versus current density characteristics, and (**d**) power efficiency versus current density characteristics of TE-OLEDs using Cr/Al/Cr, Cr/Al/Cr/TiN, and Cr/Al/Cr/Mo. The inset in (**b**) shows a typical photograph of the TE-OLED driven at 5 mA/cm^2^.

Then, we examined the electroluminescence (EL) performance of the TE-OLED devices using the Cr/Al/Cr/TiN anode in comparison with Cr/Al/Cr and Cr/Al/Cr/Mo. The devices have a general configuration of Cr (5 nm)/Al (35 nm)/Cr (5 nm)/TiN (0 and 5 nm) or Mo (5 nm)/HAT-CN (10 nm)/TAPC (160 nm)/TCTA (10 nm)/CBP: 8 wt% Ir(ppy)_2_(acac) (20 nm)/TPBi (45 nm)/Liq (2 nm)/Mg: Ag (9:1) (20 nm)/NPB (70 nm), in which HAT-CN, TAPC, TCTA, CBP, Ir(ppy)_2_(acac), TPBi, Liq, and NPB refer to 1,4,5,8,9,11-hexaazatriphenylenehexacarbonitrile, 1,1-bis[(di-4-tolylamino)phenyl]cyclohexane, tris(4-carbazoyl-9-ylphenyl)amine, 4,4′-bis(N-carbazolyl)-1,1′-biphenyl, bis [2-(2-pyridinyl-N)phenyl-C](acetylacetonate)iridium(III), 2,2′,2″-(1,3,5-Benzinetriyl)-tris(1-phenyl-1-H-benzimidazole), 8-hydroxyquinolinolato-lithium, and N,N′-Di(1-naphthyl)-N,N′-diphenyl-(1,1′-biphenyl)-4,4′-diamine, respectively. Note that NPB is a capping layer, which is reported for improving the light extraction efficiency of TE-OLEDs [38]. Figure 6c shows energy band diagram of the device, in which the values are obtained from the references [39,40]. Figure 7a compares current density–voltage–luminance characteristics of the top-emitting devices. The current density of TE-OLED with TiN is much higher than those of devices without TiN. The turn-on voltage (defined as the voltage at a current density of 0.1 mA/cm^2^) drastically decreases from 3.4 to 3.1 V when the Cr/Al/Cr anode is coated with TiN. These results indicate that the slightly increase work function upon the addition of TiN substantially reduces the hole injection barrier at the interface between Cr/Al/Cr and HTL. Shown in Figure 7c,d are the current efficiency (CE) and power efficiency (PE), respectively, of the top-emitting devices as a function of current density. The efficient hole injection from Cr/Al/Cr/TiN to TAPC greatly improves the efficiency of the TiN-containing device, with a maximum CE (CE_max_) of 71.2 cd/A and a maximum PE (PE_max_) of 66.7 lm/W, which are much higher than those of devices without TiN (39.2 cd/A and 35.1 lm/W, respectively).

As compared to Cr/Al/Cr/Mo, microcavity effects existing in the TE-OLEDs using Cr/Al/Cr/TiN may partly account for the improved EL efficiency. Figure 7b shows the normalized EL spectra of the top-emitting devices measured at a current density of 5 mA/cm^2^. The device with TiN shows a more saturated color with a narrowed EL spectrum as compared to the Mo-containing device. This could be attributed to the Cr/Al/Cr/TiN being more reflective than the Cr/Al/Cr/Mo (Figure 4), which will lead to a much stronger microcavity effect in the TE-OLED [41,42,43]. Figure 7b also shows the shoulder and peak in the TEOLED with Mo coated anode structure and the blue shifted emission peak in TEOLEDs without and with TiN coated anode. This can be attributed to the different reflectivity of the three anodes under the same optical cavity length. Different reflectivity will lead to the deviation between the interference peak of microcavity and the intrinsic luminescence peak of guest material.

By altering the thickness of TAPC, we can tune the microcavity resonance wavelengths close to the emission peak of Ir(ppy)_2_(acac) (Supplementary Figure S6). In the allowed cavity mode (second-cavity mode in our cases), the photon density of states can be spatially and spectrally redistributed such that the spontaneous emission intensity is enhanced in the forward direction relative to a noncavity device. The enhancement of the emission intensity along the cavity axis (at the resonance wavelength) is given by [44]

(1) $$G_{e}=\frac{\zeta }{2}\frac{{\left(1+\sqrt{R_{1}}\right)}^{2}\left(1-R_{2}\right)}{{\left(1-\sqrt{R_{1}R_{2}}\right)}^{2}}\frac{\tau_{cav}}{\tau },$$

where $R_{1}$ is the reflectance of the bottom anode, $R_{2}$ is the reflectance of the semi-transparent cathode, and ζ is the antinode enhancement factor, $\tau_{cav}/\tau$ is the ratio of exciton lifetimes in the cavity device and the noncavity device. Given the ζ has a maximum value of 2 and the $\tau_{cav}/\tau$ approximates to unity, the value of $R_{1}R_{2}$ could be estimated by [45]

(2) $$Q=\frac{\lambda }{\Delta \lambda }=\frac{2\pi L}{\lambda }{\left[-\ln{\left(R_{1}R_{2}\right)}^{0.5}\right]}^{-1},$$

which relates the Q of the cavity to the resonance wavelength (λ), cavity optical thickness (L), and mode linewidth (Δλ). We can clearly find that the larger enhancement in EL efficiency of the Cr/Al/Cr/TiN device relative to the Cr/Al/Cr/Mo device may partly arises from the increased reflectance of the bottom anode (i.e., the increased Q of the cavity).

## 4. Conclusions

In conclusion, we demonstrated that the TiN-coated metallic multilayers (Cr/Al/Cr/TiN) could be used as efficient reflective bottom anode to produce high-performance TE-OLEDs. The EL efficiency of the device with Cr/Al/Cr/TiN is remarkably higher than those with Cr/Al/Cr and Cr/Al/Cr/Mo. This is attributed to the slightly increased work function of Cr/Al/Cr/TiN ensuring an efficient hole injection at the anode/HTL interface and the relatively high reflectance of Cr/Al/Cr/TiN causing a strong microcavity effect in TE-OLED. Moreover, the smooth TiN layer forming on the Cr/Al/Cr anode not only protects the composite electrode from corrosion reaction with oxygen and air moisture, but also successfully improves the alkali-resistant property of the electrode. Thus, our study provides an inexpensive but promising anode, which could be used to produce high-performance and CMOS-compatible TE-OLEDs for microdisplays.

## Figures and Tables

**Table 1:** The sheet resistance of the Cr/Al/Cr/TiN and Cr/Al/Cr/Mo anodes under the ultrasonic treatment for a different time.

Sample	Sheet Resistance after Treating for Different Times (Ω/□)			
	0 s	5 s	10 s	15 s
Cr/Al/Cr/TiN	8.8	9.0	9.2	11.2
Cr/Al/Cr/Mo	3.4	5.4	7.6	7.7

## Data Availability

Data is contained within the article or Supplementary Materials.

## Supplementary Materials

The following supporting information can be downloaded at: https://www.mdpi.com/article/10.3390/molecules27175723/s1, Figure S1: Atomic force microscope (AFM) images of the Cr/Al/Cr/TiN anodes with different thicknesses of TiN; Figure S2: Atomic force microscope (AFM) images of the Cr/Al/Cr/TiN anodes with different thicknesses of TiN; Figure S3: SEM images of the Cr/Al/Cr/TiN anode under the ultrasonic treatment for a different time; Figure S4: SEM images of the Cr/Al/Cr/Mo anode under the ultrasonic treatment for a different time; Figure S5: Electroluminescence spectra of Cr/Al/Cr/TiN-based TE-OLEDs using different TAPC thicknesses. Figure S6: Electroluminescence spectra of Cr/Al/Cr/TiN-based TE-OLEDs using different TAPC thicknesses.

## Sample Availability

Samples of the compounds are not available from the authors.
