# Supplementary material for: Robust Laminated Anode with an Ultrathin Titanium Nitride Layer for High-Efficiency Top-Emitting Organic Light-Emitting Diodes
Source: Molecules. 2022 Sep 5;27(17):5723. doi: 10.3390/molecules27175723 (PMC9457887; doi:10.3390/molecules27175723)
Supplement: Supplementary file 1 [file molecules-27-05723-s001.zip › molecules-1867827-supplementary.pdf]

Supplementary information

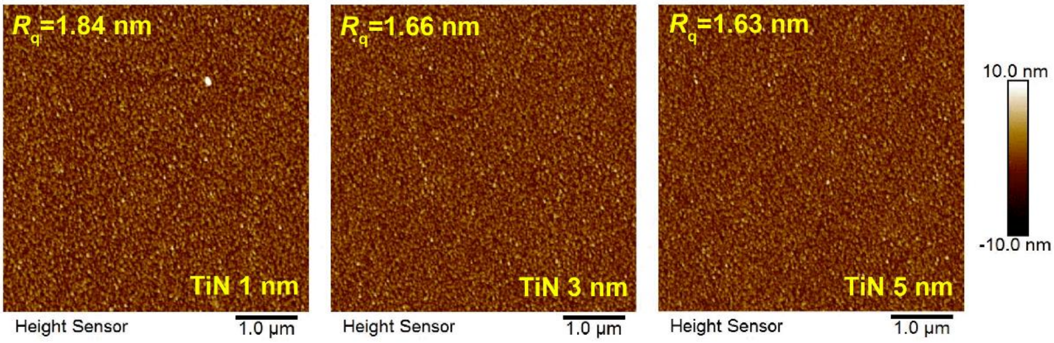

Figure S1. Atomic force microscope (AFM) images of the Cr/Al/Cr/TiN anodes with different thicknesses of TiN.

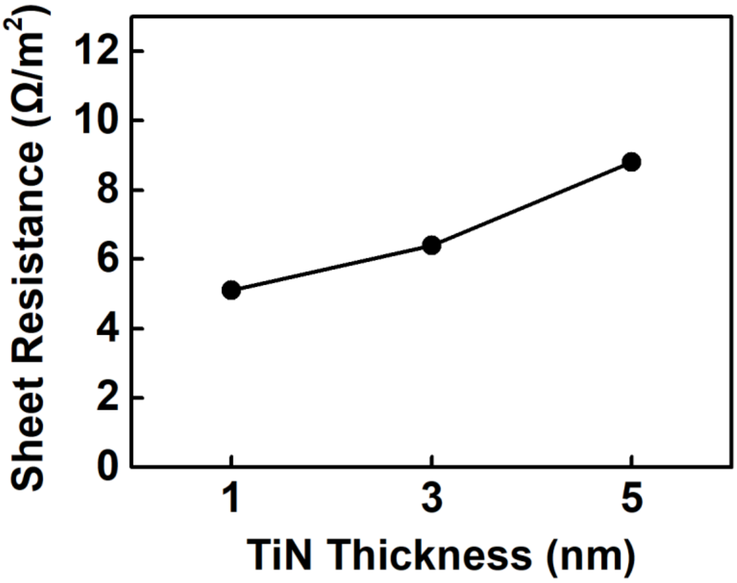

Figure S2. Atomic force microscope (AFM) images of the Cr/Al/Cr/TiN anodes with different thicknesses of TiN.

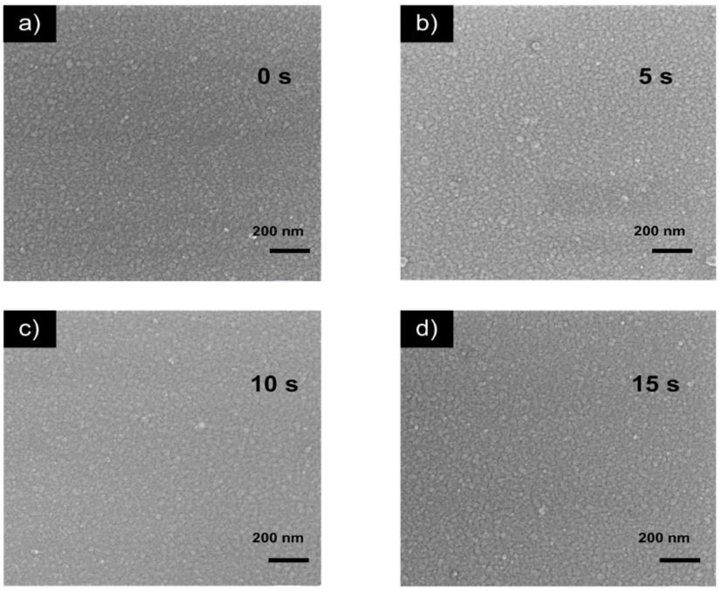

Figure S3. SEM images of the Cr/Al/Cr/TiN anode under the ultrasonic treatment for a different time.

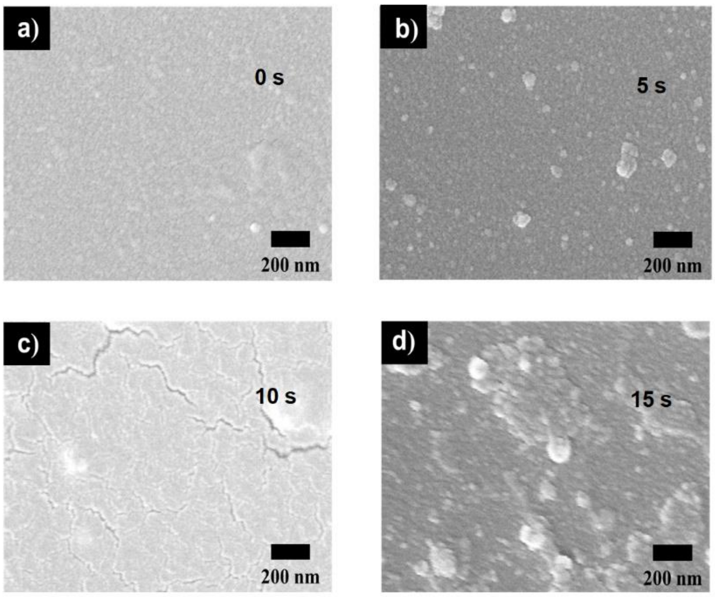

Figure S4. SEM images of the Cr/Al/Cr/Mo anode under the ultrasonic treatment for a different time.

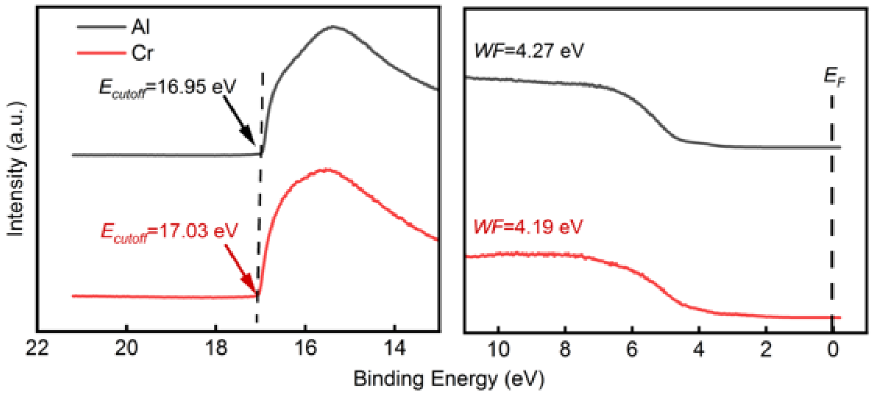

Figure S5. UPS spectra of the Al and Cr.

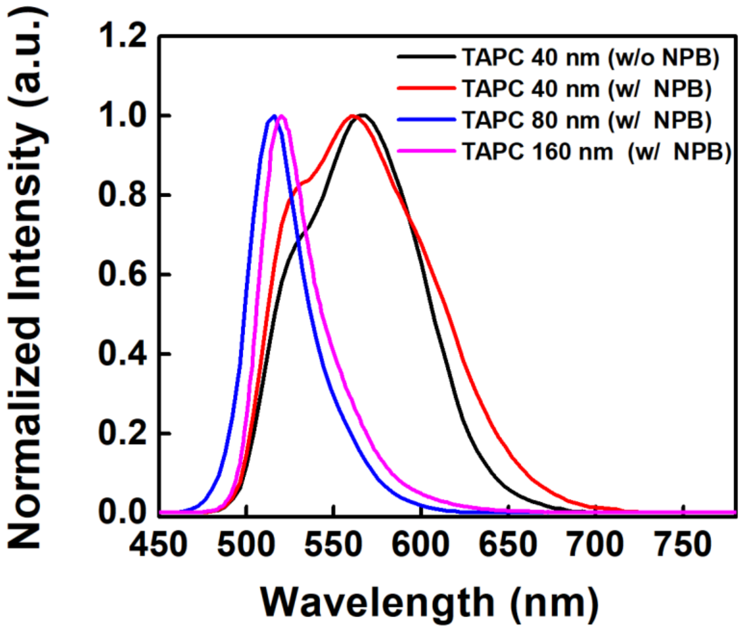

Figure S6. Electroluminescence spectra of Cr/Al/Cr/TiN-based TE-OLEDs using different TAPC thicknesses.
